# Supplementary material for: Analysis of PD-L1 and CD3 Expression in Glioblastoma Patients and Correlation with Outcome: A Single Center Report
Source: Biomedicines. 2023 Jan 22;11(2):311. doi: 10.3390/biomedicines11020311 (PMC9953166; doi:10.3390/biomedicines11020311)
Supplement: Supplementary file 1 [file biomedicines-11-00311-s001.zip › biomedicines-2108363-supplementary.pptx]

## Slide 1
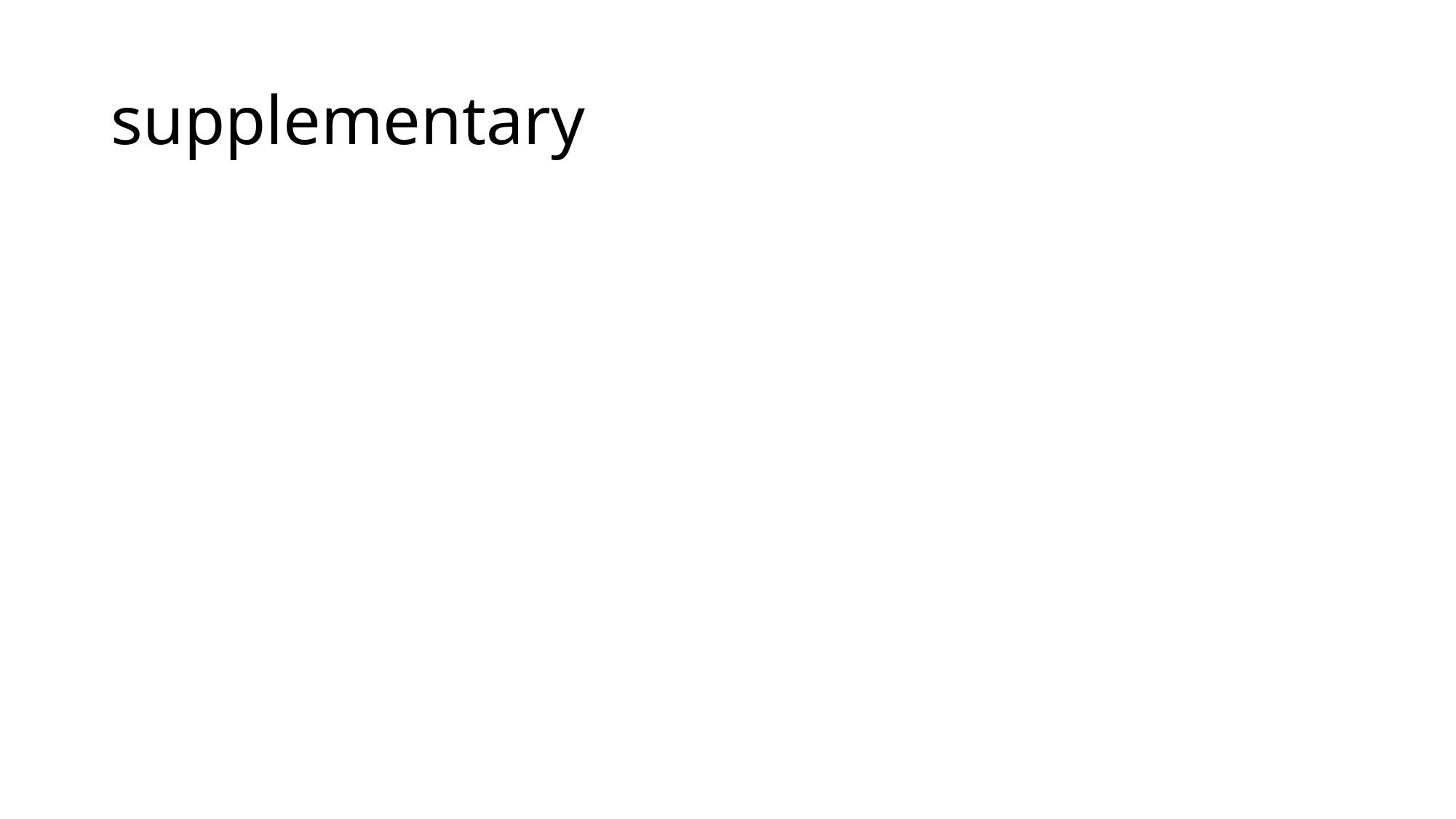

# supplementary

## Slide 2
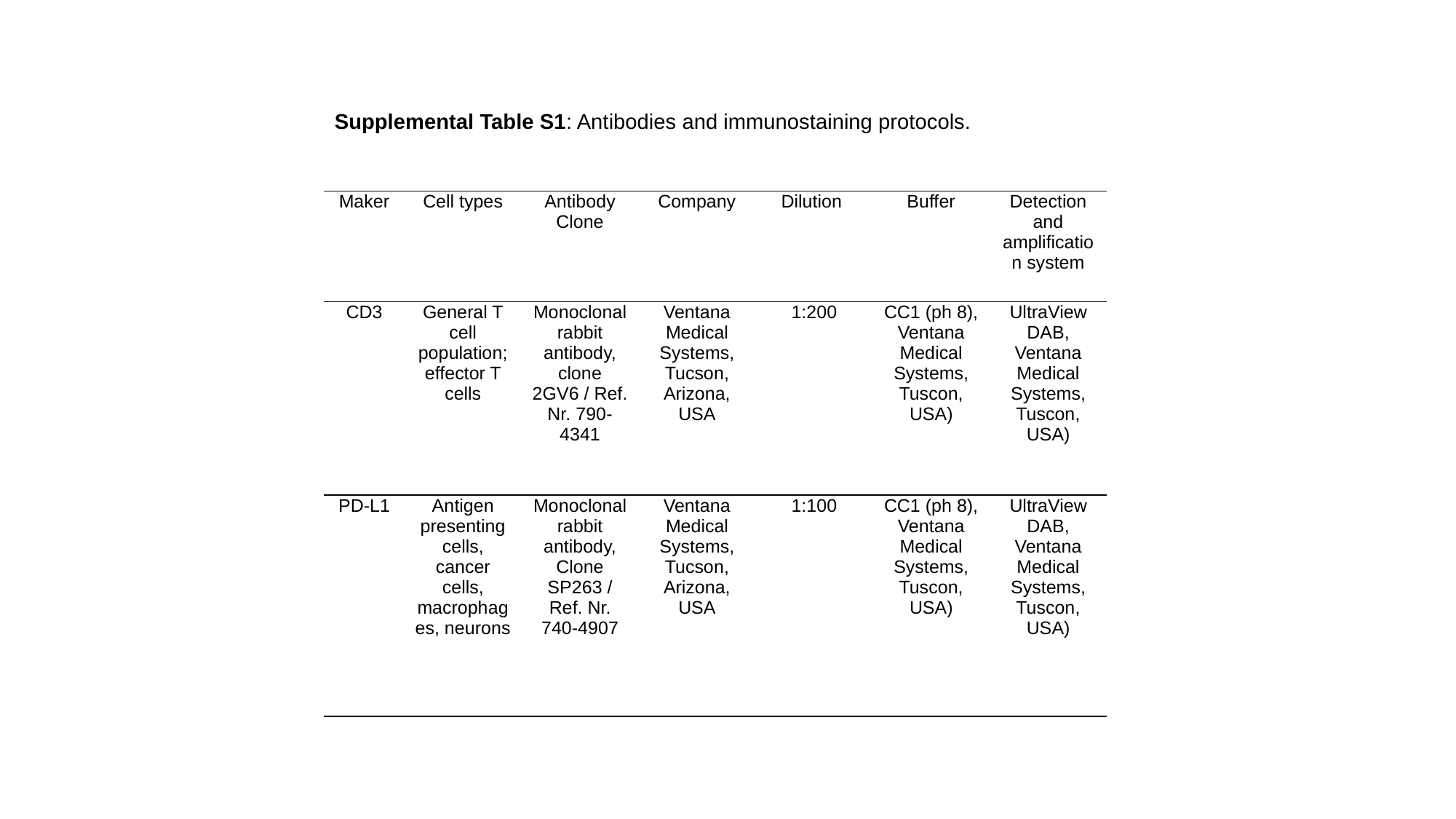

Supplemental Table S1: Antibodies and immunostaining protocols.
| Maker | Cell types | Antibody Clone | Company | Dilution | Buffer | Detection and amplification system |
| --- | --- | --- | --- | --- | --- | --- |
| CD3 | General T cell population; effector T cells | Monoclonal rabbit antibody, clone 2GV6 / Ref. Nr. 790-4341 | Ventana Medical Systems, Tucson, Arizona, USA | 1:200 | CC1 (ph 8), Ventana Medical Systems, Tuscon, USA) | UltraView DAB, Ventana Medical Systems, Tuscon, USA) |
| PD-L1 | Antigen presenting cells, cancer cells, macrophages, neurons | Monoclonal rabbit antibody, Clone SP263 / Ref. Nr. 740-4907 | Ventana Medical Systems, Tucson, Arizona, USA | 1:100 | CC1 (ph 8), Ventana Medical Systems, Tuscon, USA) | UltraView DAB, Ventana Medical Systems, Tuscon, USA) |

## Slide 3
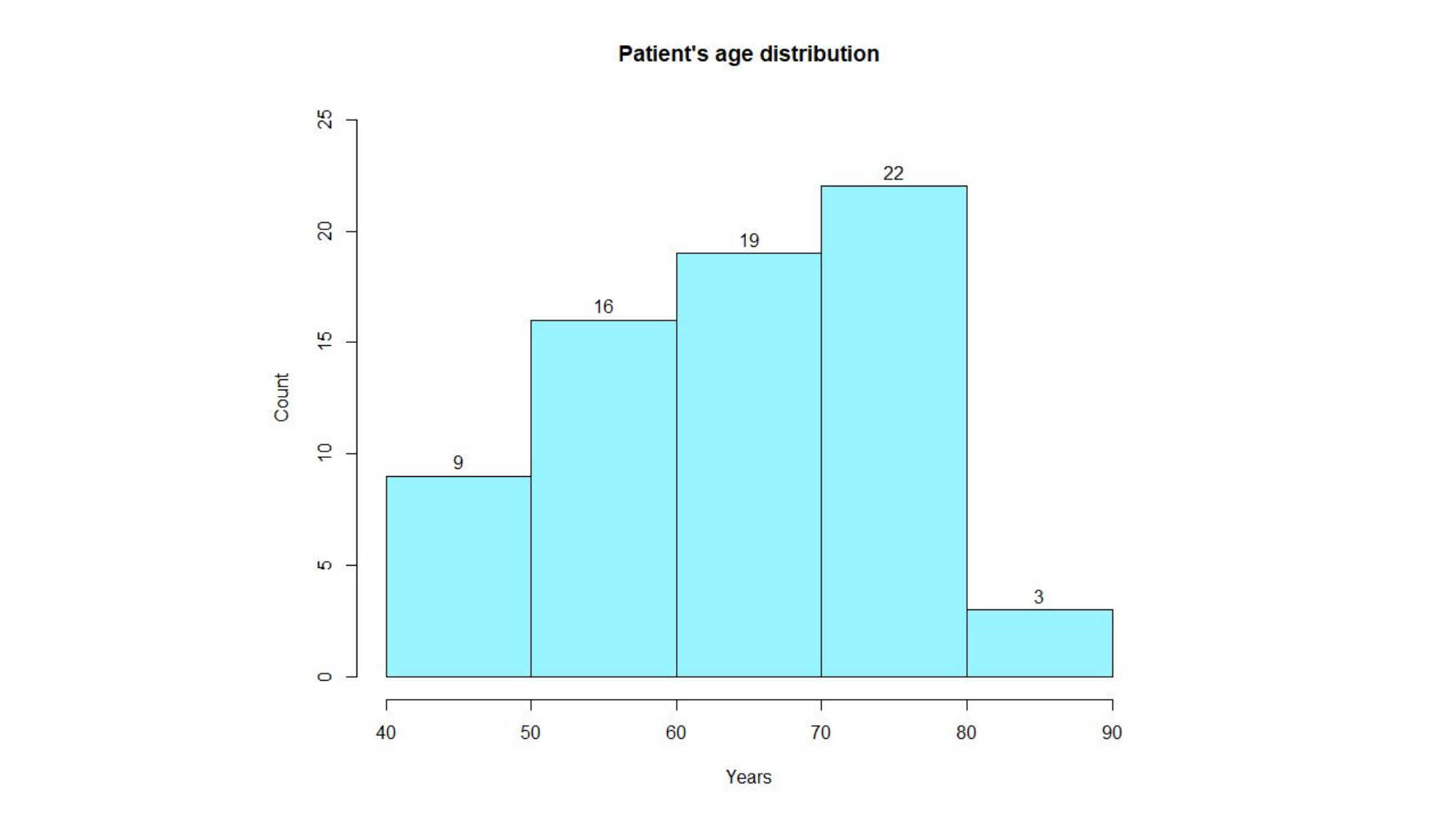

## Slide 4
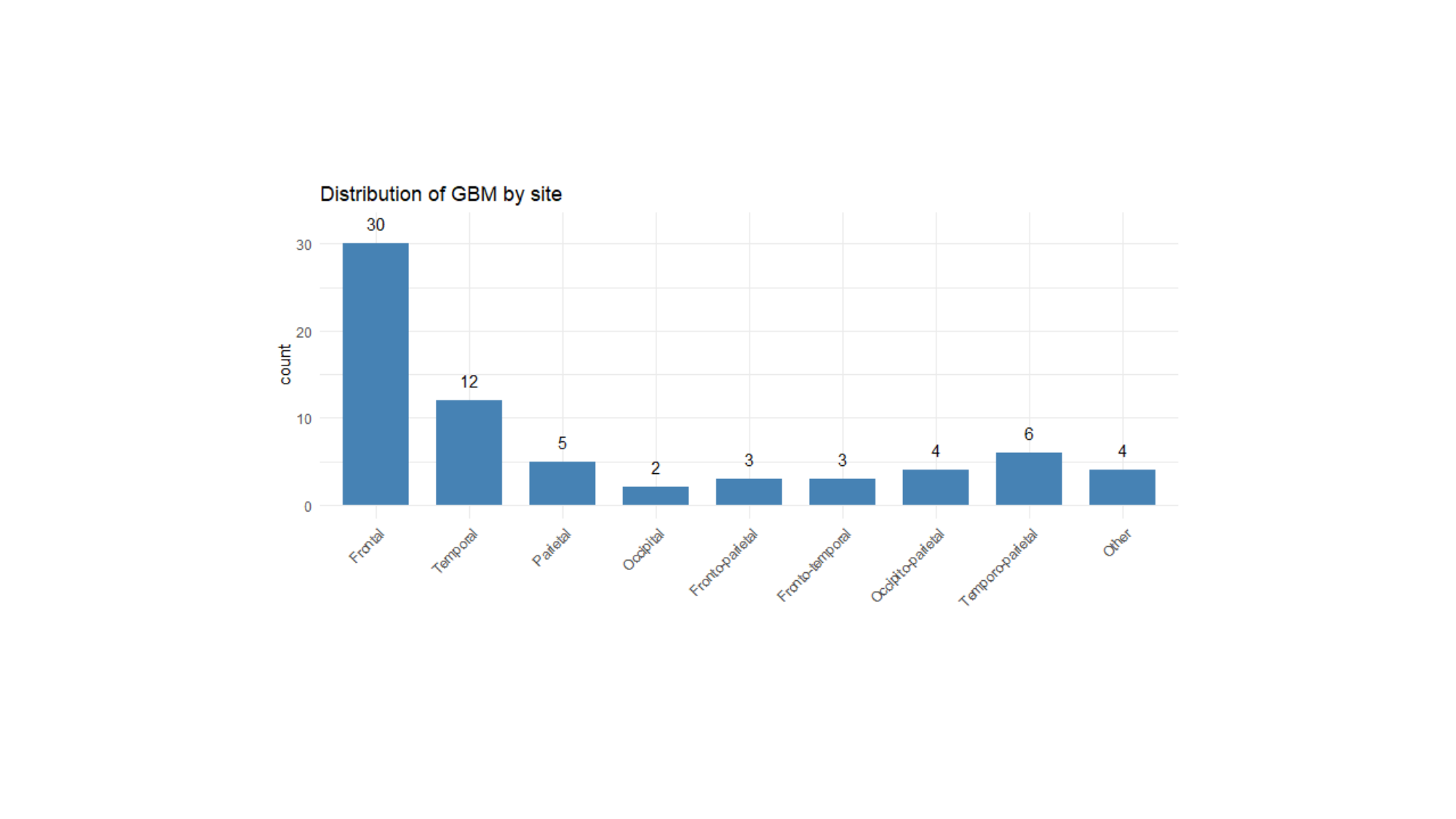

## Slide 5
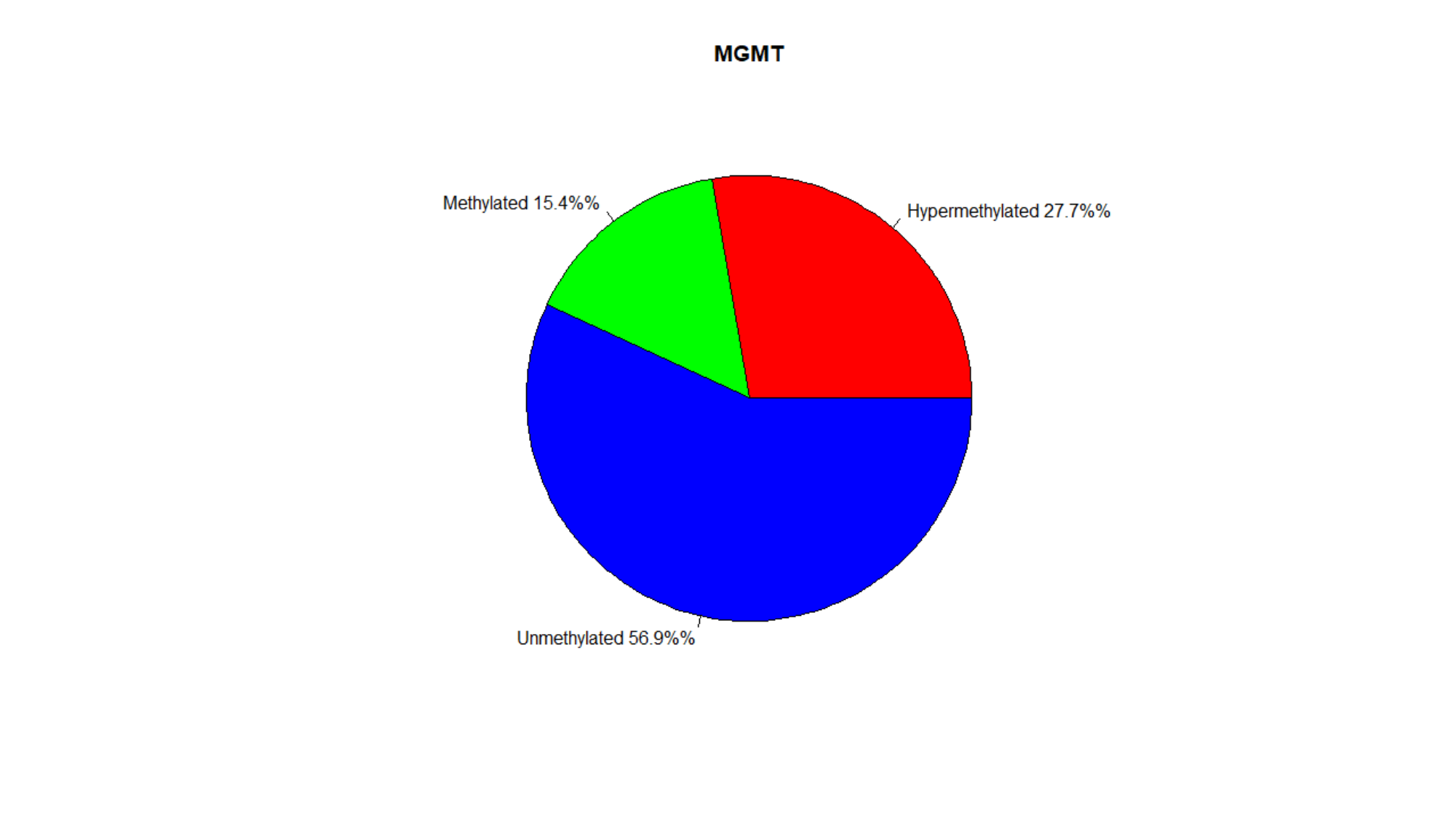

## Slide 6
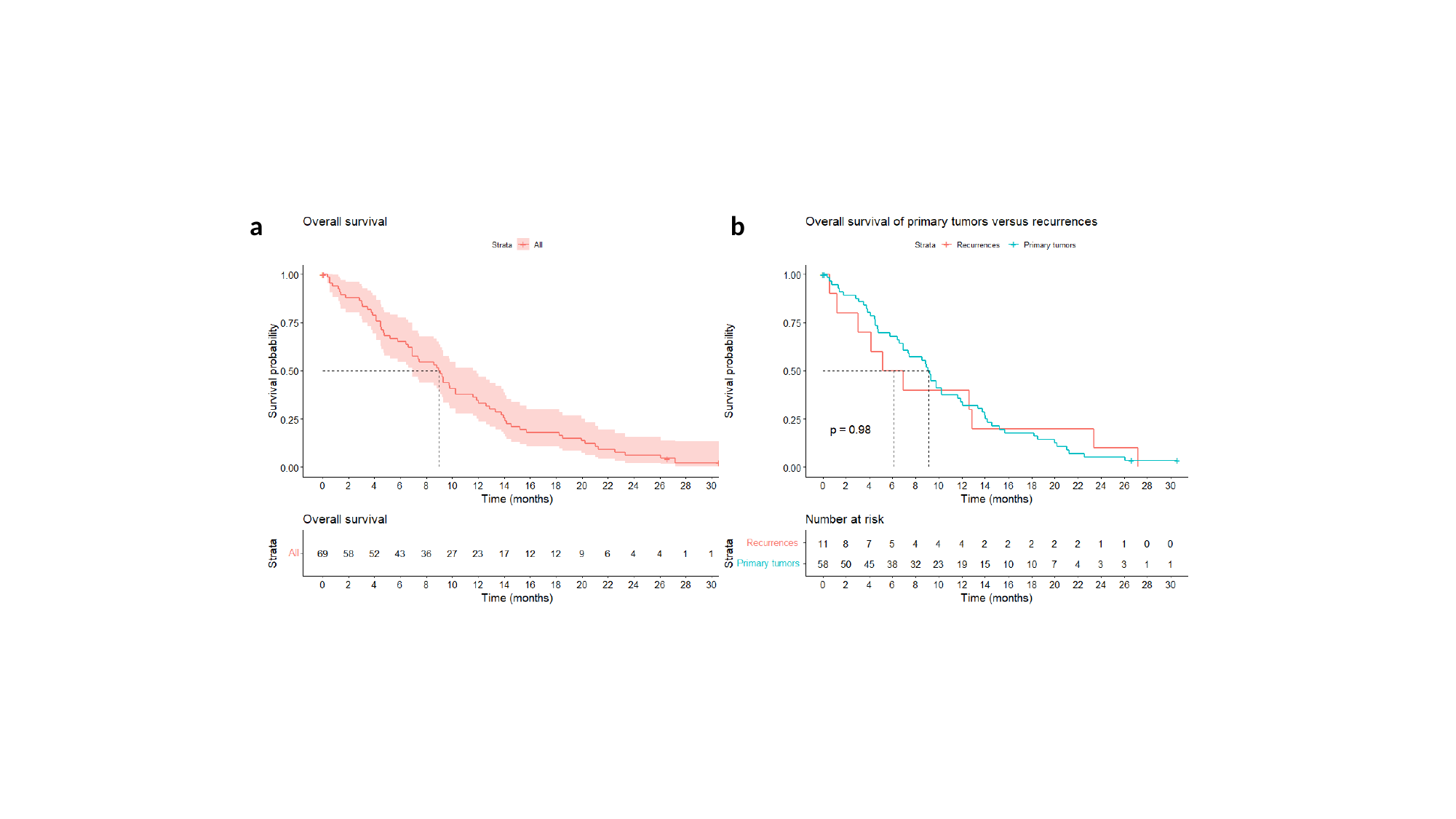

a
b
